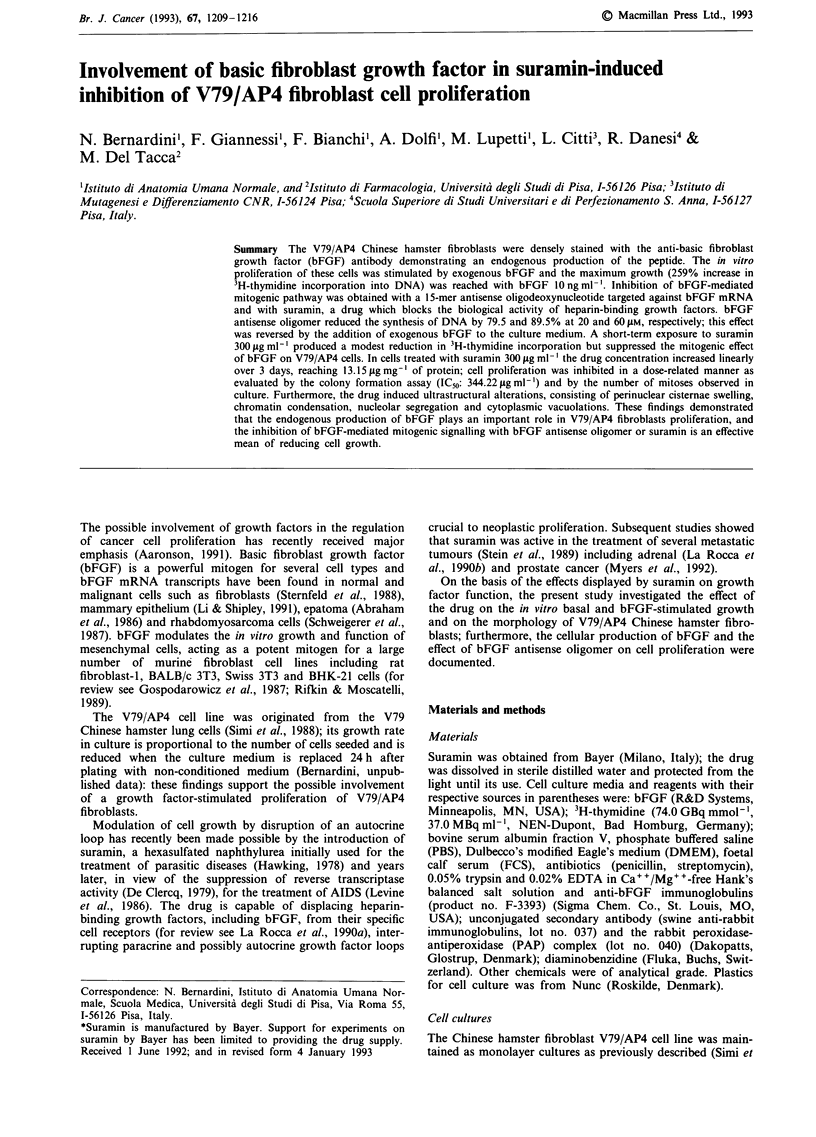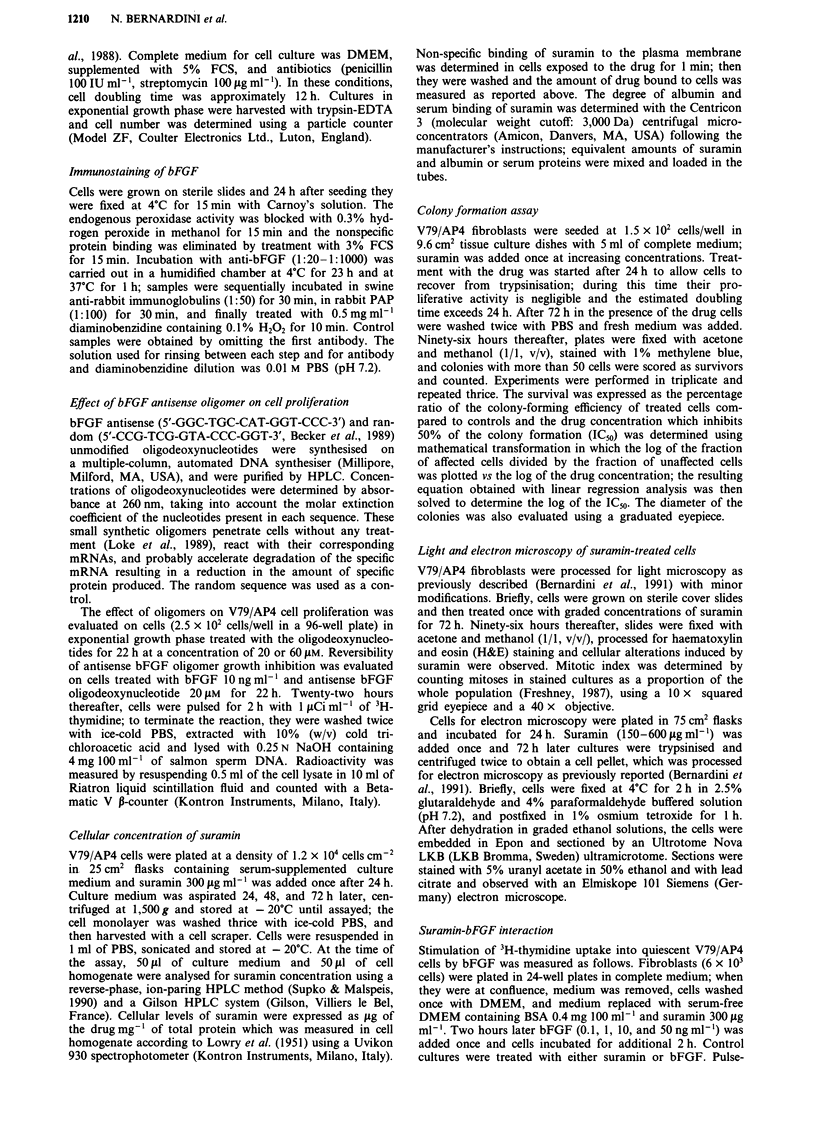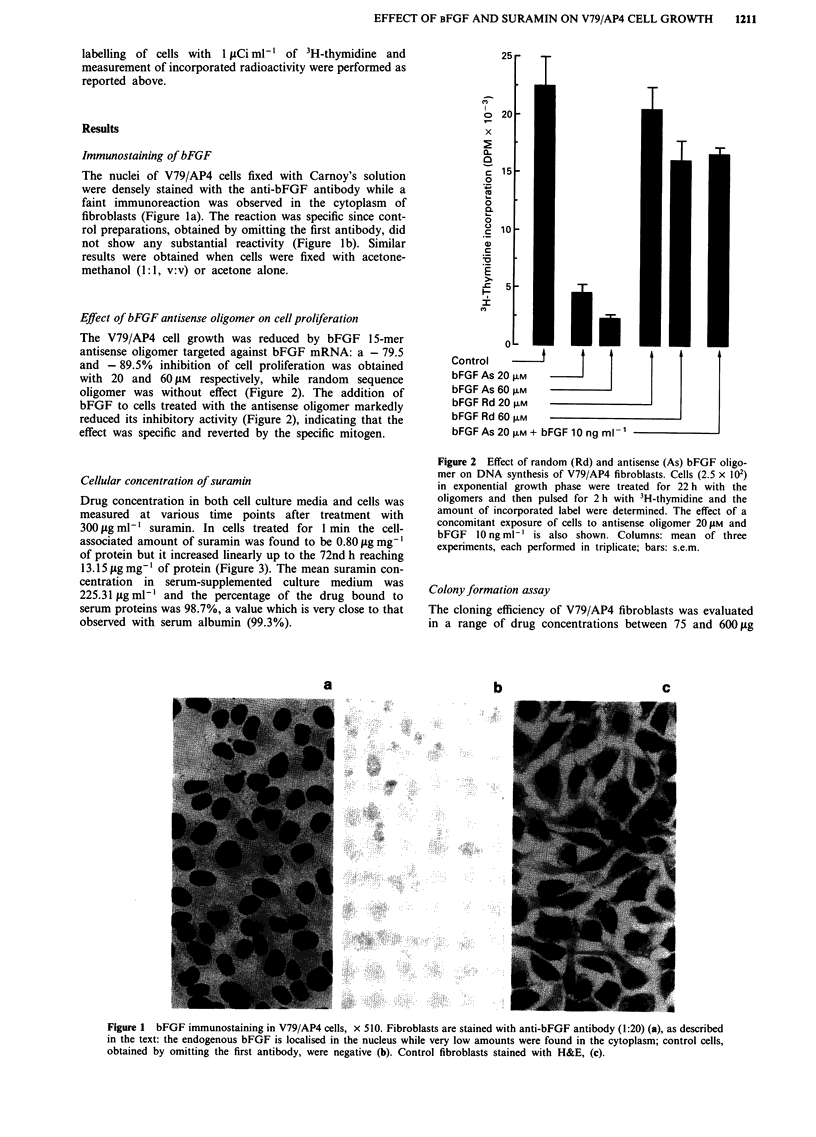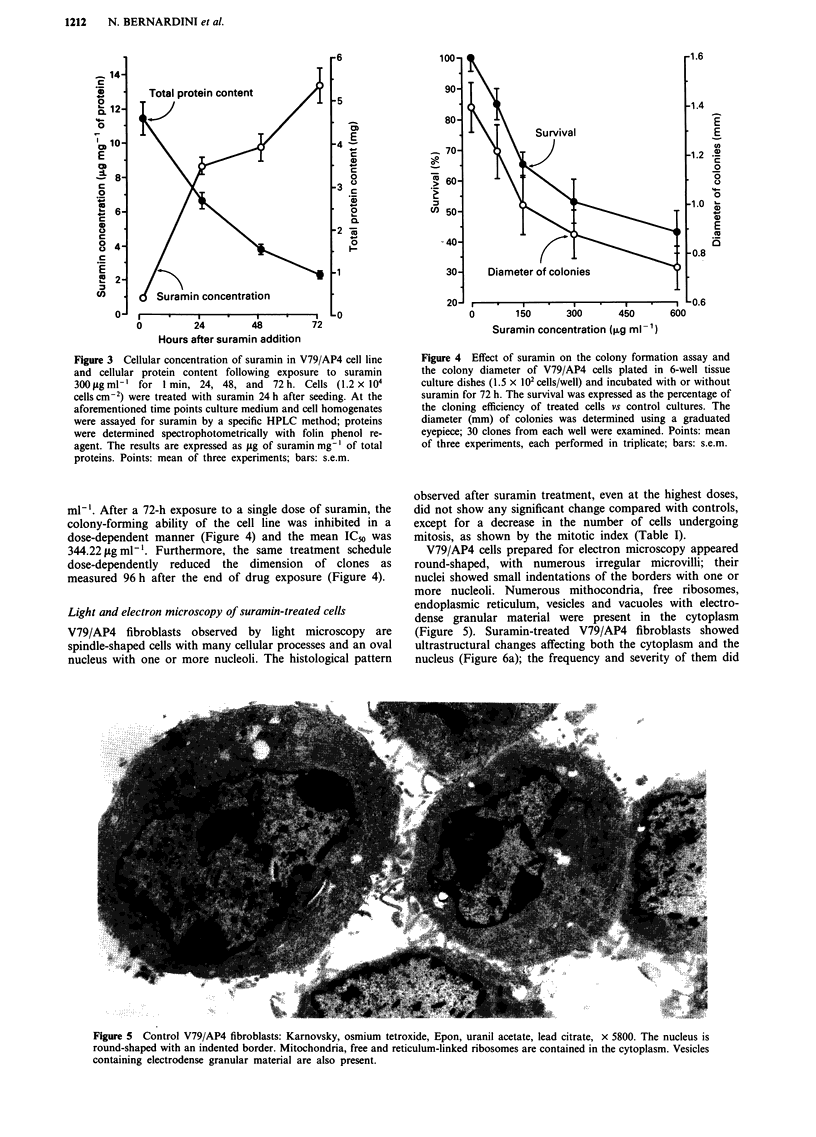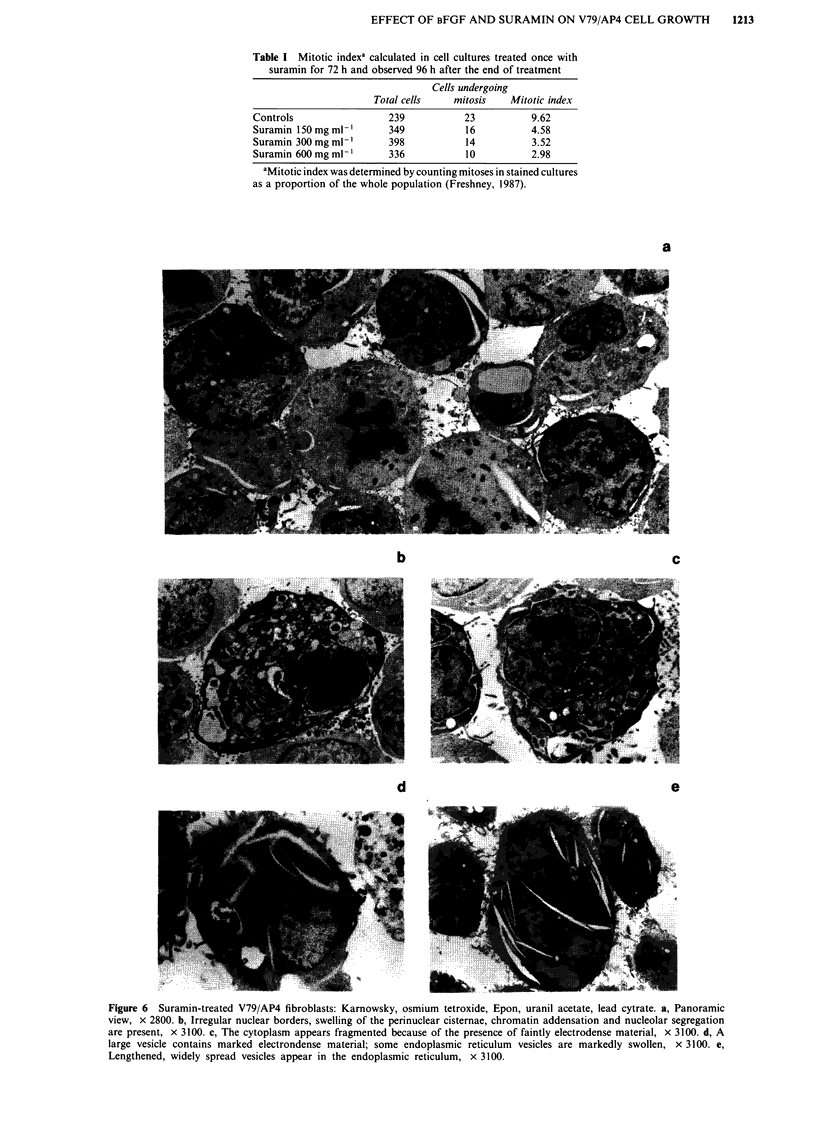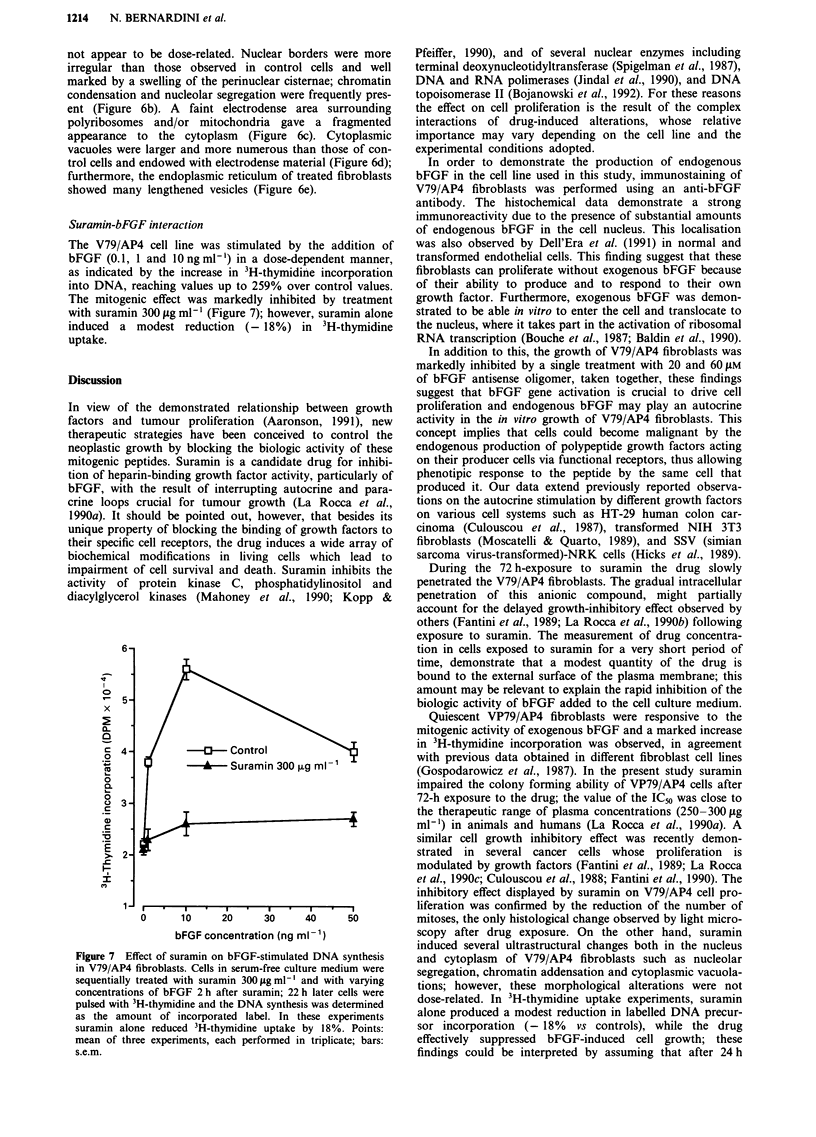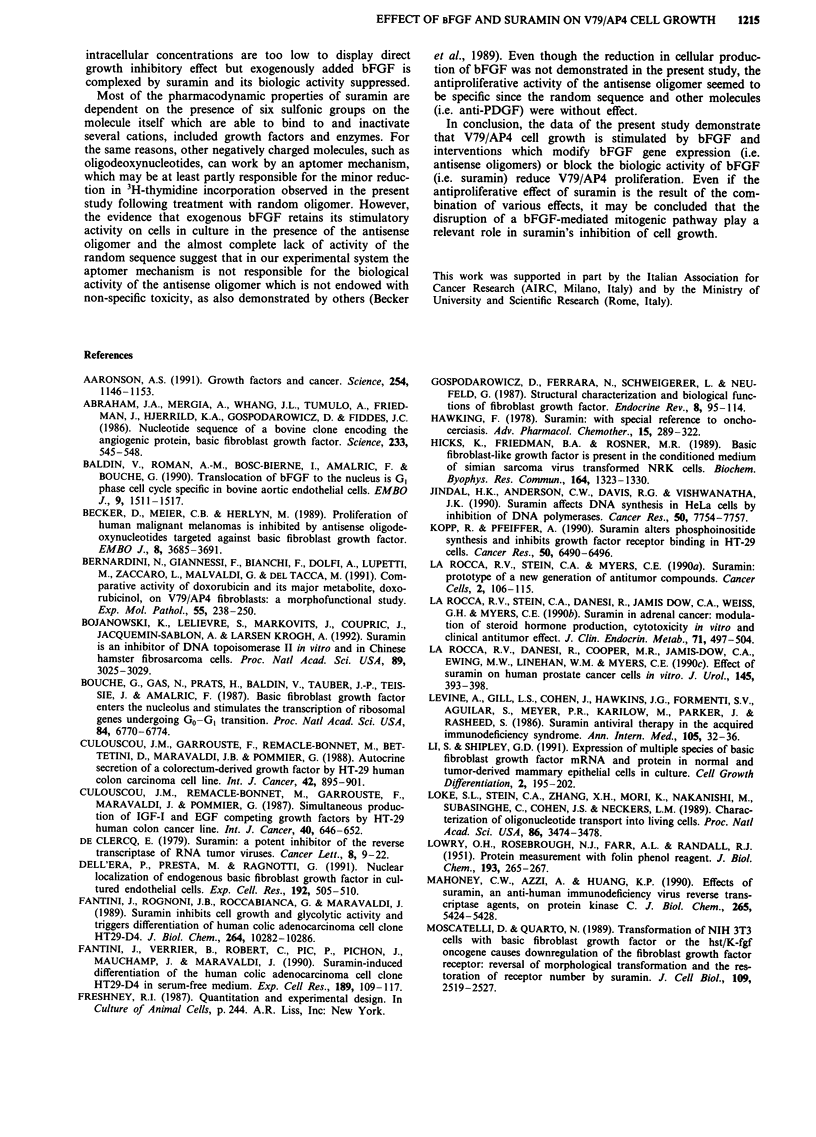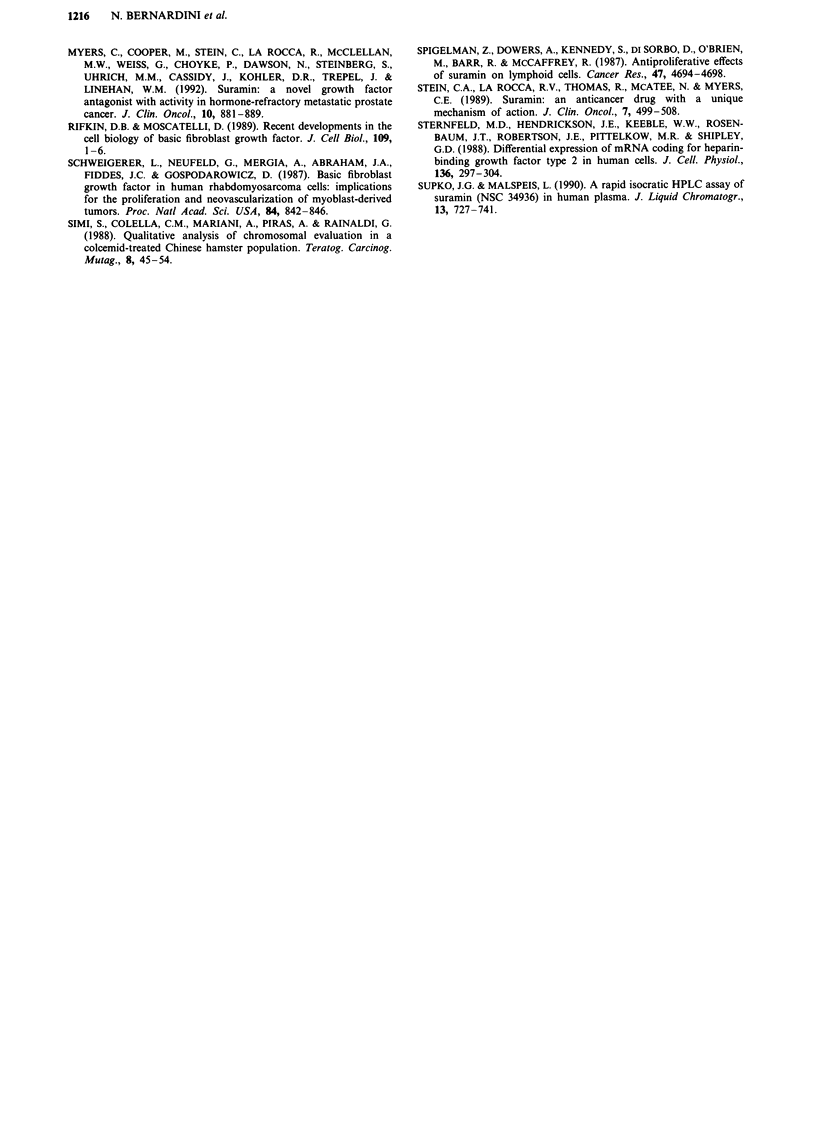# Involvement of basic fibroblast growth factor in suramin-induced inhibition of V79/AP4 fibroblast cell proliferation.

**DOI:** 10.1038/bjc.1993.227

**Published:** 1993-06

**Authors:** N. Bernardini, F. Giannessi, F. Bianchi, A. Dolfi, M. Lupetti, L. Citti, R. Danesi, M. Del Tacca

**Affiliations:** Istituto di Anatomia Umana Normale, Università degli Studi di Pisa, Italy.

## Abstract

**Images:**


Br. J. Cancer (1993), 67, 1209-1216 © Macmillan Press Ltd., 1993

### Involvement of basic fibroblast growth factor in suramin-induced inhibition of V79/AP4 fibroblast cell proliferation

N. Bernardini[1], F. Giannessi[1], F. Bianchi[1], A. Dolfi[1], M. Lupetti[1], L. Citti[3], R. Danesi[4] & M. Del Tacca[2]

[1]*Istituto di Anatomia Umana Normale, and* [2]*Istituto di Farmacologia, Università degli Studi di Pisa, I-56126 Pisa;* [3]*Istituto di Mutagenesi e Differenziamento CNR, I-56124 Pisa;* [4]*Scuola Superiore di Studi Universitari e di Perfezionamento S. Anna, I-56127 Pisa, Italy.*

**Summary** The V79/AP4 Chinese hamster fibroblasts were densely stained with the anti-basic fibroblast growth factor (bFGF) antibody demonstrating an endogenous production of the peptide. The *in vitro* proliferation of these cells was stimulated by exogenous bFGF and the maximum growth (259% increase in $^{3}$H-thymidine incorporation into DNA) was reached with bFGF 10 ng ml$^{-1}$. Inhibition of bFGF-mediated mitogenic pathway was obtained with a 15-mer antisense oligodeoxynucleotide targeted against bFGF mRNA and with suramin, a drug which blocks the biological activity of heparin-binding growth factors. bFGF antisense oligomer reduced the synthesis of DNA by 79.5 and 89.5% at 20 and 60 $\mu$M, respectively; this effect was reversed by the addition of exogenous bFGF to the culture medium. A short-term exposure to suramin 300 $\mu$g ml$^{-1}$ produced a modest reduction in $^{3}$H-thymidine incorporation but suppressed the mitogenic effect of bFGF on V79/AP4 cells. In cells treated with suramin 300 $\mu$g ml$^{-1}$ the drug concentration increased linearly over 3 days, reaching 13.15 $\mu$g mg$^{-1}$ of protein; cell proliferation was inhibited in a dose-related manner as evaluated by the colony formation assay (IC$_{50}$: 344.22 $\mu$g ml$^{-1}$) and by the number of mitoses observed in culture. Furthermore, the drug induced ultrastructural alterations, consisting of perinuclear cisternae swelling, chromatin condensation, nucleolar segregation and cytoplasmic vacuolations. These findings demonstrated that the endogenous production of bFGF plays an important role in V79/AP4 fibroblasts proliferation, and the inhibition of bFGF-mediated mitogenic signalling with bFGF antisense oligomer or suramin is an effective mean of reducing cell growth.

The possible involvement of growth factors in the regulation of cancer cell proliferation has recently received major emphasis (Aaronson, 1991). Basic fibroblast growth factor (bFGF) is a powerful mitogen for several cell types and bFGF mRNA transcripts have been found in normal and malignant cells such as fibroblasts (Sternfeld *et al.*, 1988), mammary epithelium (Li & Shipley, 1991), epatoma (Abraham *et al.*, 1986) and rhabdomyosarcoma cells (Schweigerer *et al.*, 1987). bFGF modulates the *in vitro* growth and function of mesenchymal cells, acting as a potent mitogen for a large number of murine fibroblast cell lines including rat fibroblast-1, BALB/c 3T3, Swiss 3T3 and BHK-21 cells (for review see Gospodarowicz *et al.*, 1987; Rifkin & Moscatelli, 1989).

The V79/AP4 cell line was originated from the V79 Chinese hamster lung cells (Simi *et al.*, 1988); its growth rate in culture is proportional to the number of cells seeded and is reduced when the culture medium is replaced 24 h after plating with non-conditioned medium (Bernardini, unpublished data): these findings support the possible involvement of a growth factor-stimulated proliferation of V79/AP4 fibroblasts.

Modulation of cell growth by disruption of an autocrine loop has recently been made possible by the introduction of suramin, a hexasulfated naphthylurea initially used for the treatment of parasitic diseases (Hawking, 1978) and years later, in view of the suppression of reverse transcriptase activity (De Clercq, 1979), for the treatment of AIDS (Levine *et al.*, 1986). The drug is capable of displacing heparin-binding growth factors, including bFGF, from their specific cell receptors (for review see La Rocca *et al.*, 1990*a*), interrupting paracrine and possibly autocrine growth factor loops crucial to neoplastic proliferation. Subsequent studies showed that suramin was active in the treatment of several metastatic tumours (Stein *et al.*, 1989) including adrenal (La Rocca *et al.*, 1990*b*) and prostate cancer (Myers *et al.*, 1992).

On the basis of the effects displayed by suramin on growth factor function, the present study investigated the effect of the drug on the *in vitro* basal and bFGF-stimulated growth and on the morphology of V79/AP4 Chinese hamster fibroblasts; furthermore, the cellular production of bFGF and the effect of bFGF antisense oligomer on cell proliferation were documented.

### Materials and methods

### Materials

Suramin was obtained from Bayer (Milano, Italy); the drug was dissolved in sterile distilled water and protected from the light until its use. Cell culture media and reagents with their respective sources in parentheses were: bFGF (R&D Systems, Minneapolis, MN, USA); $^{3}$H-thymidine (74.0 GBq mmol$^{-1}$, 37.0 MBq ml$^{-1}$, NEN-Dupont, Bad Homburg, Germany); bovine serum albumin fraction V, phosphate buffered saline (PBS), Dulbecco's modified Eagle's medium (DMEM), foetal calf serum (FCS), antibiotics (penicillin, streptomycin), 0.05% trypsin and 0.02% EDTA in Ca$^{++}$/Mg$^{++}$-free Hank's balanced salt solution and anti-bFGF immunoglobulins (product no. F-3393) (Sigma Chem. Co., St. Louis, MO, USA); unconjugated secondary antibody (swine anti-rabbit immunoglobulins, lot no. 037) and the rabbit peroxidase-antiperoxidase (PAP) complex (lot no. 040) (Dakopatts, Glostrup, Denmark); diaminobenzidine (Fluka, Buchs, Switzerland). Other chemicals were of analytical grade. Plastics for cell culture was from Nunc (Roskilde, Denmark).

### Cell cultures

The Chinese hamster fibroblast V79/AP4 cell line was maintained as monolayer cultures as previously described (Simi *et*

Correspondence: N. Bernardini, Istituto di Anatomia Umana Normale, Scuola Medica, Università degli Studi di Pisa, Via Roma 55, I-56126 Pisa, Italy.
*Suramin is manufactured by Bayer. Support for experiments on suramin by Bayer has been limited to providing the drug supply.
Received 1 June 1992; and in revised form 4 January 1993

al., 1988). Complete medium for cell culture was DMEM, supplemented with 5% FCS, and antibiotics (penicillin 100 IU $ml^{-1}$, streptomycin 100 μg $ml^{-1}$). In these conditions, cell doubling time was approximately 12 h. Cultures in exponential growth phase were harvested with trypsin-EDTA and cell number was determined using a particle counter (Model ZF, Coulter Electronics Ltd., Luton, England).

*Immunostaining of bFGF*

Cells were grown on sterile slides and 24 h after seeding they were fixed at 4°C for 15 min with Carnoy's solution. The endogenous peroxidase activity was blocked with 0.3% hydrogen peroxide in methanol for 15 min and the nonspecific protein binding was eliminated by treatment with 3% FCS for 15 min. Incubation with anti-bFGF (1:20–1:1000) was carried out in a humidified chamber at 4°C for 23 h and at 37°C for 1 h; samples were sequentially incubated in swine anti-rabbit immunoglobulins (1:50) for 30 min, in rabbit PAP (1:100) for 30 min, and finally treated with 0.5 mg $ml^{-1}$ diaminobenzidine containing 0.1% $H_2O_2$ for 10 min. Control samples were obtained by omitting the first antibody. The solution used for rinsing between each step and for antibody and diaminobenzidine dilution was 0.01 M PBS (pH 7.2).

*Effect of bFGF antisense oligomer on cell proliferation*

bFGF antisense (5'-GGC-TGC-CAT-GGT-CCC-3') and random (5'-CCG-TCG-GTA-CCC-GGT-3', Becker et al., 1989) unmodified oligodeoxynucleotides were synthesised on a multiple-column, automated DNA synthesiser (Millipore, Milford, MA, USA), and were purified by HPLC. Concentrations of oligodeoxynucleotides were determined by absorbance at 260 nm, taking into account the molar extinction coefficient of the nucleotides present in each sequence. These small synthetic oligomers penetrate cells without any treatment (Loke et al., 1989), react with their corresponding mRNAs, and probably accelerate degradation of the specific mRNA resulting in a reduction in the amount of specific protein produced. The random sequence was used as a control.

The effect of oligomers on V79/AP4 cell proliferation was evaluated on cells ($2.5 \times 10^2$ cells/well in a 96-well plate) in exponential growth phase treated with the oligodeoxynucleotides for 22 h at a concentration of 20 or 60 μM. Reversibility of antisense bFGF oligomer growth inhibition was evaluated on cells treated with bFGF 10 ng $ml^{-1}$ and antisense bFGF oligodeoxynucleotide 20 μM for 22 h. Twenty-two hours thereafter, cells were pulsed for 2 h with 1 μCi $ml^{-1}$ of $^3$H-thymidine; to terminate the reaction, they were washed twice with ice-cold PBS, extracted with 10% (w/v) cold trichloroacetic acid and lysed with 0.25 N NaOH containing 4 mg 100 $ml^{-1}$ of salmon sperm DNA. Radioactivity was measured by resuspending 0.5 ml of the cell lysate in 10 ml of Riatron liquid scintillation fluid and counted with a Betamatic V β-counter (Kontron Instruments, Milano, Italy).

*Cellular concentration of suramin*

V79/AP4 cells were plated at a density of $1.2 \times 10^4$ cells $cm^{-2}$ in 25 $cm^2$ flasks containing serum-supplemented culture medium and suramin 300 μg $ml^{-1}$ was added once after 24 h. Culture medium was aspirated 24, 48, and 72 h later, centrifuged at 1,500 **g** and stored at – 20°C until assayed; the cell monolayer was washed thrice with ice-cold PBS, and then harvested with a cell scraper. Cells were resuspended in 1 ml of PBS, sonicated and stored at – 20°C. At the time of the assay, 50 μl of culture medium and 50 μl of cell homogenate were analysed for suramin concentration using a reverse-phase, ion-paring HPLC method (Supko & Malspeis, 1990) and a Gilson HPLC system (Gilson, Villiers le Bel, France). Cellular levels of suramin were expressed as μg of the drug $mg^{-1}$ of total protein which was measured in cell homogenate according to Lowry et al. (1951) using a Uvikon 930 spectrophotometer (Kontron Instruments, Milano, Italy).

Non-specific binding of suramin to the plasma membrane was determined in cells exposed to the drug for 1 min; then they were washed and the amount of drug bound to cells was measured as reported above. The degree of albumin and serum binding of suramin was determined with the Centricon 3 (molecular weight cutoff: 3,000 Da) centrifugal microconcentrators (Amicon, Danvers, MA, USA) following the manufacturer's instructions; equivalent amounts of suramin and albumin or serum proteins were mixed and loaded in the tubes.

*Colony formation assay*

V79/AP4 fibroblasts were seeded at $1.5 \times 10^2$ cells/well in 9.6 $cm^2$ tissue culture dishes with 5 ml of complete medium; suramin was added once at increasing concentrations. Treatment with the drug was started after 24 h to allow cells to recover from trypsinisation; during this time their proliferative activity is negligible and the estimated doubling time exceeds 24 h. After 72 h in the presence of the drug cells were washed twice with PBS and fresh medium was added. Ninety-six hours thereafter, plates were fixed with acetone and methanol (1/1, v/v), stained with 1% methylene blue, and colonies with more than 50 cells were scored as survivors and counted. Experiments were performed in triplicate and repeated thrice. The survival was expressed as the percentage ratio of the colony-forming efficiency of treated cells compared to controls and the drug concentration which inhibits 50% of the colony formation ($IC_{50}$) was determined using mathematical transformation in which the log of the fraction of affected cells divided by the fraction of unaffected cells was plotted *vs* the log of the drug concentration; the resulting equation obtained with linear regression analysis was then solved to determine the log of the $IC_{50}$. The diameter of the colonies was also evaluated using a graduated eyepiece.

*Light and electron microscopy of suramin-treated cells*

V79/AP4 fibroblasts were processed for light microscopy as previously described (Bernardini et al., 1991) with minor modifications. Briefly, cells were grown on sterile cover slides and then treated once with graded concentrations of suramin for 72 h. Ninety-six hours thereafter, slides were fixed with acetone and methanol (1/1, v/v/), processed for haematoxylin and eosin (H&E) staining and cellular alterations induced by suramin were observed. Mitotic index was determined by counting mitoses in stained cultures as a proportion of the whole population (Freshney, 1987), using a 10 × squared grid eyepiece and a 40 × objective.

Cells for electron microscopy were plated in 75 $cm^2$ flasks and incubated for 24 h. Suramin (150–600 μg $ml^{-1}$) was added once and 72 h later cultures were trypsinised and centrifuged twice to obtain a cell pellet, which was processed for electron microscopy as previously reported (Bernardini et al., 1991). Briefly, cells were fixed at 4°C for 2 h in 2.5% glutaraldehyde and 4% paraformaldehyde buffered solution (pH 7.2), and postfixed in 1% osmium tetroxide for 1 h. After dehydration in graded ethanol solutions, the cells were embedded in Epon and sectioned by an Ultrotome Nova LKB (LKB Bromma, Sweden) ultramicrotome. Sections were stained with 5% uranyl acetate in 50% ethanol and with lead citrate and observed with an Elmiskope 101 Siemens (Germany) electron microscope.

*Suramin-bFGF interaction*

Stimulation of $^3$H-thymidine uptake into quiescent V79/AP4 cells by bFGF was measured as follows. Fibroblasts ($6 \times 10^3$ cells) were plated in 24-well plates in complete medium; when they were at confluence, medium was removed, cells washed once with DMEM, and medium replaced with serum-free DMEM containing BSA 0.4 mg 100 $ml^{-1}$ and suramin 300 μg $ml^{-1}$. Two hours later bFGF (0.1, 1, 10, and 50 ng $ml^{-1}$) was added once and cells incubated for additional 2 h. Control cultures were treated with either suramin or bFGF. Pulselabelling of cells with 1 μCi $ml^{-1}$ of $^3$H-thymidine and measurement of incorporated radioactivity were performed as reported above.

### Results

### *Immunostaining of bFGF*

The nuclei of V79/AP4 cells fixed with Carnoy's solution were densely stained with the anti-bFGF antibody while a faint immunoreaction was observed in the cytoplasm of fibroblasts (Figure 1a). The reaction was specific since control preparations, obtained by omitting the first antibody, did not show any substantial reactivity (Figure 1b). Similar results were obtained when cells were fixed with acetone-methanol (1:1, v:v) or acetone alone.

### *Effect of bFGF antisense oligomer on cell proliferation*

The V79/AP4 cell growth was reduced by bFGF 15-mer antisense oligomer targeted against bFGF mRNA: a − 79.5 and − 89.5% inhibition of cell proliferation was obtained with 20 and 60 μM respectively, while random sequence oligomer was without effect (Figure 2). The addition of bFGF to cells treated with the antisense oligomer markedly reduced its inhibitory activity (Figure 2), indicating that the effect was specific and reverted by the specific mitogen.

### *Cellular concentration of suramin*

Drug concentration in both cell culture media and cells was measured at various time points after treatment with 300 μg $ml^{-1}$ suramin. In cells treated for 1 min the cell-associated amount of suramin was found to be 0.80 μg $mg^{-1}$ of protein but it increased linearly up to the 72nd h reaching 13.15 μg $mg^{-1}$ of protein (Figure 3). The mean suramin concentration in serum-supplemented culture medium was 225.31 μg $ml^{-1}$ and the percentage of the drug bound to serum proteins was 98.7%, a value which is very close to that observed with serum albumin (99.3%).

**Figure 2** Effect of random (Rd) and antisense (As) bFGF oligomer on DNA synthesis of V79/AP4 fibroblasts. Cells (2.5 × $10^2$) in exponential growth phase were treated for 22 h with the oligomers and then pulsed for 2 h with $^3$H-thymidine and the amount of incorporated label were determined. The effect of a concomitant exposure of cells to antisense oligomer 20 μM and bFGF 10 ng $ml^{-1}$ is also shown. Columns: mean of three experiments, each performed in triplicate; bars: s.e.m.

### *Colony formation assay*

The cloning efficiency of V79/AP4 fibroblasts was evaluated in a range of drug concentrations between 75 and 600 μg

**Figure 1** bFGF immunostaining in V79/AP4 cells, × 510. Fibroblasts are stained with anti-bFGF antibody (1:20) (**a**), as described in the text: the endogenous bFGF is localised in the nucleus while very low amounts were found in the cytoplasm; control cells, obtained by omitting the first antibody, were negative (**b**). Control fibroblasts stained with H&E, (**c**).

**Figure 3** Cellular concentration of suramin in V79/AP4 cell line and cellular protein content following exposure to suramin 300 $\mu$g ml$^{-1}$ for 1 min, 24, 48, and 72 h. Cells ($1.2 \times 10^4$ cells cm$^{-2}$) were treated with suramin 24 h after seeding. At the aforementioned time points culture medium and cell homogenates were assayed for suramin by a specific HPLC method; proteins were determined spectrophotometrically with folin phenol reagent. The results are expressed as $\mu$g of suramin mg$^{-1}$ of total proteins. Points: mean of three experiments; bars: s.e.m.

**Figure 4** Effect of suramin on the colony formation assay and the colony diameter of V79/AP4 cells plated in 6-well tissue culture dishes ($1.5 \times 10^2$ cells/well) and incubated with or without suramin for 72 h. The survival was expressed as the percentage of the cloning efficiency of treated cells *vs* control cultures. The diameter (mm) of colonies was determined using a graduated eyepiece; 30 clones from each well were examined. Points: mean of three experiments, each performed in triplicate; bars: s.e.m.

ml$^{-1}$. After a 72-h exposure to a single dose of suramin, the colony-forming ability of the cell line was inhibited in a dose-dependent manner (Figure 4) and the mean $IC_{50}$ was 344.22 $\mu$g ml$^{-1}$. Furthermore, the same treatment schedule dose-dependently reduced the dimension of clones as measured 96 h after the end of drug exposure (Figure 4).

### *Light and electron microscopy of suramin-treated cells*

V79/AP4 fibroblasts observed by light microscopy are spindle-shaped cells with many cellular processes and an oval nucleus with one or more nucleoli. The histological pattern observed after suramin treatment, even at the highest doses, did not show any significant change compared with controls, except for a decrease in the number of cells undergoing mitosis, as shown by the mitotic index (Table I).

V79/AP4 cells prepared for electron microscopy appeared round-shaped, with numerous irregular microvilli; their nuclei showed small indentations of the borders with one or more nucleoli. Numerous mithocondria, free ribosomes, endoplasmic reticulum, vesicles and vacuoles with electrodense granular material were present in the cytoplasm (Figure 5). Suramin-treated V79/AP4 fibroblasts showed ultrastructural changes affecting both the cytoplasm and the nucleus (Figure 6a); the frequency and severity of them did

**Figure 5** Control V79/AP4 fibroblasts: Karnovsky, osmium tetroxide, Epon, uranil acetate, lead citrate, × 5800. The nucleus is round-shaped with an indented border. Mitochondria, free and reticulum-linked ribosomes are contained in the cytoplasm. Vesicles containing electrodense granular material are also present.

**Table I** Mitotic index[a] calculated in cell cultures treated once with suramin for 72 h and observed 96 h after the end of treatment

| | *Total cells* | *Cells undergoing mitosis* | *Mitotic index* |
|---|---|---|---|
| Controls | 239 | 23 | 9.62 |
| Suramin 150 mg ml$^{-1}$ | 349 | 16 | 4.58 |
| Suramin 300 mg ml$^{-1}$ | 398 | 14 | 3.52 |
| Suramin 600 mg ml$^{-1}$ | 336 | 10 | 2.98 |

[a]Mitotic index was determined by counting mitoses in stained cultures as a proportion of the whole population (Freshney, 1987).

**Figure 6** Suramin-treated V79/AP4 fibroblasts: Karnowsky, osmium tetroxide, Epon, uranil acetate, lead cytrate. **a**, Panoramic view, × 2800. **b**, Irregular nuclear borders, swelling of the perinuclear cisternae, chromatin addensation and nucleolar segregation are present, × 3100. **c**, The cytoplasm appears fragmented because of the presence of faintly electrodense material, × 3100. **d**, A large vesicle contains marked electrondense material; some endoplasmic reticulum vesicles are markedly swollen, × 3100. **e**, Lengthened, widely spread vesicles appear in the endoplasmic reticulum, × 3100.

not appear to be dose-related. Nuclear borders were more irregular than those observed in control cells and well marked by a swelling of the perinuclear cisternae; chromatin condensation and nucleolar segregation were frequently present (Figure 6b). A faint electrodense area surrounding polyribosomes and/or mitochondria gave a fragmented appearance to the cytoplasm (Figure 6c). Cytoplasmic vacuoles were larger and more numerous than those of control cells and endowed with electrodense material (Figure 6d); furthermore, the endoplasmic reticulum of treated fibroblasts showed many lengthened vesicles (Figure 6e).

*Suramin-bFGF interaction*

The V79/AP4 cell line was stimulated by the addition of bFGF (0.1, 1 and 10 ng ml$^{-1}$) in a dose-dependent manner, as indicated by the increase in $^3$H-thymidine incorporation into DNA, reaching values up to 259% over control values. The mitogenic effect was markedly inhibited by treatment with suramin 300 µg ml$^{-1}$ (Figure 7); however, suramin alone induced a modest reduction (– 18%) in $^3$H-thymidine uptake.

### Discussion

In view of the demonstrated relationship between growth factors and tumour proliferation (Aaronson, 1991), new therapeutic strategies have been conceived to control the neoplastic growth by blocking the biologic activity of these mitogenic peptides. Suramin is a candidate drug for inhibition of heparin-binding growth factor activity, particularly of bFGF, with the result of interrupting autocrine and paracrine loops crucial for tumour growth (La Rocca *et al.*, 1990*a*). It should be pointed out, however, that besides its unique property of blocking the binding of growth factors to their specific cell receptors, the drug induces a wide array of biochemical modifications in living cells which lead to impairment of cell survival and death. Suramin inhibits the activity of protein kinase C, phosphatidylinositol and diacylglycerol kinases (Mahoney *et al.*, 1990; Kopp & Pfeiffer, 1990), and of several nuclear enzymes including terminal deoxynucleotidyltransferase (Spigelman *et al.*, 1987), DNA and RNA polimerases (Jindal *et al.*, 1990), and DNA topoisomerase II (Bojanowski *et al.*, 1992). For these reasons the effect on cell proliferation is the result of the complex interactions of drug-induced alterations, whose relative importance may vary depending on the cell line and the experimental conditions adopted.

**Figure 7** Effect of suramin on bFGF-stimulated DNA synthesis in V79/AP4 fibroblasts. Cells in serum-free culture medium were sequentially treated with suramin 300 µg ml$^{-1}$ and with varying concentrations of bFGF 2 h after suramin; 22 h later cells were pulsed with $^3$H-thymidine and the DNA synthesis was determined as the amount of incorporated label. In these experiments suramin alone reduced $^3$H-thymidine uptake by 18%. Points: mean of three experiments, each performed in triplicate; bars: s.e.m.

In order to demonstrate the production of endogenous bFGF in the cell line used in this study, immunostaining of V79/AP4 fibroblasts was performed using an anti-bFGF antibody. The histochemical data demonstrate a strong immunoreactivity due to the presence of substantial amounts of endogenous bFGF in the cell nucleus. This localisation was also observed by Dell'Era *et al.* (1991) in normal and transformed endothelial cells. This finding suggest that these fibroblasts can proliferate without exogenous bFGF because of their ability to produce and to respond to their own growth factor. Furthermore, exogenous bFGF was demonstrated to be able *in vitro* to enter the cell and translocate to the nucleus, where it takes part in the activation of ribosomal RNA transcription (Bouche *et al.*, 1987; Baldin *et al.*, 1990).

In addition to this, the growth of V79/AP4 fibroblasts was markedly inhibited by a single treatment with 20 and 60 µM of bFGF antisense oligomer, taken together, these findings suggest that bFGF gene activation is crucial to drive cell proliferation and endogenous bFGF may play an autocrine activity in the *in vitro* growth of V79/AP4 fibroblasts. This concept implies that cells could become malignant by the endogenous production of polypeptide growth factors acting on their producer cells via functional receptors, thus allowing phenotipic response to the peptide by the same cell that produced it. Our data extend previously reported observations on the autocrine stimulation by different growth factors on various cell systems such as HT-29 human colon carcinoma (Culouscou *et al.*, 1987), transformed NIH 3T3 fibroblasts (Moscatelli & Quarto, 1989), and SSV (simian sarcoma virus-transformed)-NRK cells (Hicks *et al.*, 1989).

During the 72 h-exposure to suramin the drug slowly penetrated the V79/AP4 fibroblasts. The gradual intracellular penetration of this anionic compound, might partially account for the delayed growth-inhibitory effect observed by others (Fantini *et al.*, 1989; La Rocca *et al.*, 1990*b*) following exposure to suramin. The measurement of drug concentration in cells exposed to suramin for a very short period of time, demonstrate that a modest quantity of the drug is bound to the external surface of the plasma membrane; this amount may be relevant to explain the rapid inhibition of the biologic activity of bFGF added to the cell culture medium.

Quiescent VP79/AP4 fibroblasts were responsive to the mitogenic activity of exogenous bFGF and a marked increase in $^3$H-thymidine incorporation was observed, in agreement with previous data obtained in different fibroblast cell lines (Gospodarowicz *et al.*, 1987). In the present study suramin impaired the colony forming ability of VP79/AP4 cells after 72-h exposure to the drug; the value of the $IC_{50}$ was close to the therapeutic range of plasma concentrations (250–300 µg ml$^{-1}$) in animals and humans (La Rocca *et al.*, 1990*a*). A similar cell growth inhibitory effect was recently demonstrated in several cancer cells whose proliferation is modulated by growth factors (Fantini *et al.*, 1989; La Rocca *et al.*, 1990*c*; Culouscou *et al.*, 1988; Fantini *et al.*, 1990). The inhibitory effect displayed by suramin on V79/AP4 cell proliferation was confirmed by the reduction of the number of mitoses, the only histological change observed by light microscopy after drug exposure. On the other hand, suramin induced several ultrastructural changes both in the nucleus and cytoplasm of V79/AP4 fibroblasts such as nucleolar segregation, chromatin addensation and cytoplasmic vacuolations; however, these morphological alterations were not dose-related. In $^3$H-thymidine uptake experiments, suramin alone produced a modest reduction in labelled DNA precursor incorporation (– 18% *vs* controls), while the drug effectively suppressed bFGF-induced cell growth; these findings could be interpreted by assuming that after 24 h intracellular concentrations are too low to display direct growth inhibitory effect but exogenously added bFGF is complexed by suramin and its biologic activity suppressed.

Most of the pharmacodynamic properties of suramin are dependent on the presence of six sulfonic groups on the molecule itself which are able to bind to and inactivate several cations, included growth factors and enzymes. For the same reasons, other negatively charged molecules, such as oligodeoxynucleotides, can work by an aptomer mechanism, which may be at least partly responsible for the minor reduction in $^3$H-thymidine incorporation observed in the present study following treatment with random oligomer. However, the evidence that exogenous bFGF retains its stimulatory activity on cells in culture in the presence of the antisense oligomer and the almost complete lack of activity of the random sequence suggest that in our experimental system the aptomer mechanism is not responsible for the biological activity of the antisense oligomer which is not endowed with non-specific toxicity, as also demonstrated by others (Becker et al., 1989). Even though the reduction in cellular production of bFGF was not demonstrated in the present study, the antiproliferative activity of the antisense oligomer seemed to be specific since the random sequence and other molecules (i.e. anti-PDGF) were without effect.

In conclusion, the data of the present study demonstrate that V79/AP4 cell growth is stimulated by bFGF and interventions which modify bFGF gene expression (i.e. antisense oligomers) or block the biologic activity of bFGF (i.e. suramin) reduce V79/AP4 proliferation. Even if the antiproliferative effect of suramin is the result of the combination of various effects, it may be concluded that the disruption of a bFGF-mediated mitogenic pathway play a relevant role in suramin's inhibition of cell growth.

This work was supported in part by the Italian Association for Cancer Research (AIRC, Milano, Italy) and by the Ministry of University and Scientific Research (Rome, Italy).

### References

AARONSON, A.S. (1991). Growth factors and cancer. *Science*, **254**, 1146–1153.

ABRAHAM, J.A., MERGIA, A., WHANG, J.L., TUMULO, A., FRIEDMAN, J., HJERRILD, K.A., GOSPODAROWICZ, D. & FIDDES, J.C. (1986). Nucleotide sequence of a bovine clone encoding the angiogenic protein, basic fibroblast growth factor. *Science*, **233**, 545–548.

BALDIN, V., ROMAN, A.-M., BOSC-BIERNE, I., AMALRIC, F. & BOUCHE, G. (1990). Translocation of bFGF to the nucleus is $G_1$ phase cell cycle specific in bovine aortic endothelial cells. *EMBO J.*, **9**, 1511–1517.

BECKER, D., MEIER, C.B. & HERLYN, M. (1989). Proliferation of human malignant melanomas is inhibited by antisense oligodeoxynucleotides targeted against basic fibroblast growth factor. *EMBO J.*, **8**, 3685–3691.

BERNARDINI, N., GIANNESSI, F., BIANCHI, F., DOLFI, A., LUPETTI, M., ZACCARO, L., MALVALDI, G. & DEL TACCA, M. (1991). Comparative activity of doxorubicin and its major metabolite, doxorubicinol, on V79/AP4 fibroblasts: a morphofunctional study. *Exp. Mol. Pathol.*, **55**, 238–250.

BOJANOWSKI, K., LELIEVRE, S., MARKOVITS, J., COUPRIC, J., JACQUEMIN-SABLON, A. & LARSEN KROGH, A. (1992). Suramin is an inhibitor of DNA topoisomerase II *in vitro* and in Chinese hamster fibrosarcoma cells. *Proc. Natl Acad. Sci. USA*, **89**, 3025–3029.

BOUCHE, G., GAS, N., PRATS, H., BALDIN, V., TAUBER, J.-P., TEISSIE, J. & AMALRIC, F. (1987). Basic fibroblast growth factor enters the nucleolus and stimulates the transcription of ribosomal genes undergoing $G_0$–$G_1$ transition. *Proc. Natl Acad. Sci. USA*, **84**, 6770–6774.

CULOUSCOU, J.M., GARROUSTE, F., REMACLE-BONNET, M., BETTETINI, D., MARAVALDI, J.B. & POMMIER, G. (1988). Autocrine secretion of a colorectum-derived growth factor by HT-29 human colon carcinoma cell line. *Int. J. Cancer*, **42**, 895–901.

CULOUSCOU, J.M., REMACLE-BONNET, M., GARROUSTE, F., MARAVALDI, J. & POMMIER, G. (1987). Simultaneous production of IGF-I and EGF competing growth factors by HT-29 human colon cancer line. *Int. J. Cancer*, **40**, 646–652.

DE CLERCQ, E. (1979). Suramin: a potent inhibitor of the reverse transcriptase of RNA tumor viruses. *Cancer Lett.*, **8**, 9–22.

DELL'ERA, P., PRESTA, M. & RAGNOTTI, G. (1991). Nuclear localization of endogenous basic fibroblast growth factor in cultured endothelial cells. *Exp. Cell. Res.*, **192**, 505–510.

FANTINI, J., ROGNONI, J.B., ROCCABIANCA, G. & MARAVALDI, J. (1989). Suramin inhibits cell growth and glycolytic activity and triggers differentiation of human colic adenocarcinoma cell clone HT29-D4. *J. Biol. Chem.*, **264**, 10282–10286.

FANTINI, J., VERRIER, B., ROBERT, C., PIC, P., PICHON, J., MAUCHAMP, J. & MARAVALDI, J. (1990). Suramin-induced differentiation of the human colic adenocarcinoma cell clone HT29-D4 in serum-free medium. *Exp. Cell Res.*, **189**, 109–117.

FRESHNEY, R.I. (1987). Quantitation and experimental design. In *Culture of Animal Cells*, p. 244. A.R. Liss, Inc: New York.

GOSPODAROWICZ, D., FERRARA, N., SCHWEIGERER, L. & NEUFELD, G. (1987). Structural characterization and biological functions of fibroblast growth factor. *Endocrine Rev.*, **8**, 95–114.

HAWKING, F. (1978). Suramin: with special reference to onchocerciasis. *Adv. Pharmacol. Chemother.*, **15**, 289–322.

HICKS, K., FRIEDMAN, B.A. & ROSNER, M.R. (1989). Basic fibroblast-like growth factor is present in the conditioned medium of simian sarcoma virus transformed NRK cells. *Biochem. Byophys. Res. Commun.*, **164**, 1323–1330.

JINDAL, H.K., ANDERSON, C.W., DAVIS, R.G. & VISHWANATHA, J.K. (1990). Suramin affects DNA synthesis in HeLa cells by inhibition of DNA polymerases. *Cancer Res.*, **50**, 7754–7757.

KOPP, R. & PFEIFFER, A. (1990). Suramin alters phosphoinositide synthesis and inhibits growth factor receptor binding in HT-29 cells. *Cancer Res.*, **50**, 6490–6496.

LA ROCCA, R.V., STEIN, C.A. & MYERS, C.E. (1990*a*). Suramin: prototype of a new generation of antitumor compounds. *Cancer Cells*, **2**, 106–115.

LA ROCCA, R.V., STEIN, C.A., DANESI, R., JAMIS DOW, C.A., WEISS, G.H. & MYERS, C.E. (1990*b*). Suramin in adrenal cancer: modulation of steroid hormone production, cytotoxicity *in vitro* and clinical antitumor effect. *J. Clin. Endocrin. Metab.*, **71**, 497–504.

LA ROCCA, R.V., DANESI, R., COOPER, M.R., JAMIS-DOW, C.A., EWING, M.W., LINEHAN, W.M. & MYERS, C.E. (1990*c*). Effect of suramin on human prostate cancer cells *in vitro*. *J. Urol.*, **145**, 393–398.

LEVINE, A., GILL, L.S., COHEN, J., HAWKINS, J.G., FORMENTI, S.V., AGUILAR, S., MEYER, P.R., KARILOW, M., PARKER, J. & RASHEED, S. (1986). Suramin antiviral therapy in the acquired immunodeficiency syndrome. *Ann. Intern. Med.*, **105**, 32–36.

LI, S. & SHIPLEY, G.D. (1991). Expression of multiple species of basic fibroblast growth factor mRNA and protein in normal and tumor-derived mammary epithelial cells in culture. *Cell Growth Differentiation*, **2**, 195–202.

LOKE, S.L., STEIN, C.A., ZHANG, X.H., MORI, K., NAKANISHI, M., SUBASINGHE, C., COHEN, J.S. & NECKERS, L.M. (1989). Characterization of oligonucleotide transport into living cells. *Proc. Natl Acad. Sci. USA*, **86**, 3474–3478.

LOWRY, O.H., ROSEBROUGH, N.J., FARR, A.L. & RANDALL, R.J. (1951). Protein measurement with folin phenol reagent. *J. Biol. Chem.*, **193**, 265–267.

MAHONEY, C.W., AZZI, A. & HUANG, K.P. (1990). Effects of suramin, an anti-human immunodeficiency virus reverse transcriptase agents, on protein kinase C. *J. Biol. Chem.*, **265**, 5424–5428.

MOSCATELLI, D. & QUARTO, N. (1989). Transformation of NIH 3T3 cells with basic fibroblast growth factor or the hst/K-fgf oncogene causes downregulation of the fibroblast growth factor receptor: reversal of morphological transformation and the restoration of receptor number by suramin. *J. Cell Biol.*, **109**, 2519–2527.

MYERS, C., COOPER, M., STEIN, C., LA ROCCA, R., MCCLELLAN, M.W., WEISS, G., CHOYKE, P., DAWSON, N., STEINBERG, S., UHRICH, M.M., CASSIDY, J., KOHLER, D.R., TREPEL, J. & LINEHAN, W.M. (1992). Suramin: a novel growth factor antagonist with activity in hormone-refractory metastatic prostate cancer. *J. Clin. Oncol.*, **10**, 881-889.

RIFKIN, D.B. & MOSCATELLI, D. (1989). Recent developments in the cell biology of basic fibroblast growth factor. *J. Cell Biol.*, **109**, 1-6.

SCHWEIGERER, L., NEUFELD, G., MERGIA, A., ABRAHAM, J.A., FIDDES, J.C. & GOSPODAROWICZ, D. (1987). Basic fibroblast growth factor in human rhabdomyosarcoma cells: implications for the proliferation and neovascularization of myoblast-derived tumors. *Proc. Natl Acad. Sci. USA*, **84**, 842-846.

SIMI, S., COLELLA, C.M., MARIANI, A., PIRAS, A. & RAINALDI, G. (1988). Qualitative analysis of chromosomal evaluation in a colcemid-treated Chinese hamster population. *Teratog. Carcinog. Mutag.*, **8**, 45-54.

SPIGELMAN, Z., DOWERS, A., KENNEDY, S., DI SORBO, D., O'BRIEN, M., BARR, R. & MCCAFFREY, R. (1987). Antiproliferative effects of suramin on lymphoid cells. *Cancer Res.*, **47**, 4694-4698.

STEIN, C.A., LA ROCCA, R.V., THOMAS, R., MCATEE, N. & MYERS, C.E. (1989). Suramin: an anticancer drug with a unique mechanism of action. *J. Clin. Oncol.*, **7**, 499-508.

STERNFELD, M.D., HENDRICKSON, J.E., KEEBLE, W.W., ROSENBAUM, J.T., ROBERTSON, J.E., PITTELKOW, M.R. & SHIPLEY, G.D. (1988). Differential expression of mRNA coding for heparin-binding growth factor type 2 in human cells. *J. Cell. Physiol.*, **136**, 297-304.

SUPKO, J.G. & MALSPEIS, L. (1990). A rapid isocratic HPLC assay of suramin (NSC 34936) in human plasma. *J. Liquid Chromatogr.*, **13**, 727-741.